# Dental pulp stem cells regenerate neural tissue in degenerative disorders and stroke rehabilitation: A scope systematic review

**DOI:** 10.1016/j.heliyon.2024.e35080

**Published:** 2024-07-25

**Authors:** Ali Rahnama Sisakht, Zahra Tavasouli, Ahmad Negahi, Seyed Alireza Hosseini, Mohammad Satarzadeh

**Affiliations:** a ormozgan University of Medical Sciences, Bandar Abbas, Iran; b Ghaemieh Health Care Center, Mazandaran University of Medical Sciences, Sari, Iran; c School of Medicine, Hormozgan University of Medical Sciences, Bandar Abbas, Iran; d School of Nursing and Midwifery, Yasuj University of Medical Sciences, Yasuj, Iran

**Keywords:** Dental pulp stem cells, Systematic review, Stroke, Neuroinflammation, Regeneration

## Abstract

**Background:**

Dental Pulp Stem Cells (DPSCs) possess a remarkable ability for tissue differentiation, making them highly efficient in tissue regeneration and inflammation regulation. This systematic study proposes to find an answer to the question, “Do DPSCs have the ability to regenerate and rehabilitate nerve tissue?"

**Methods:**

This systematic review was conducted based on Preferred Reporting Items for Systematic Reviews and Meta-Analyses (PRISMA) criteria, and the principle of non-bias was respected. All the articles from 2014 to 2024 were extracted from the Web of Science, PubMed, and Scopus databases. This study extracted the antigens and pro-inflammatory factors associated with DPSCs' involvement and how they affect the CNS's neural tissue regeneration.

**Results:**

Two persons of researchers searched the database. After screening the full texts, they included 11 articles in their study. DPSCs control the following antigens: CD73, CD34, CD90, CD105, CD14, CD45, CD19Oct-4, CD73, CD31, CD34CD29CD44. Even though hematopoietic markers did not change much, OCT-4 and CD-73 were increased by DPSCs. DPSC-derived exosomes suppressed the expression of IL-6, IL-1β, TNF-α, and TGF, key mediators of nerve tissue inflammation. Additionally, DPSCs show high Vascular Endothelial Growth Factor (VEGF) expression in mice brain tissue cultures. DPSCs reduce Subarachnoid Hemorrhage (SAH), a condition in which blood collects in the subarachnoid space and causes ischemia.

**Discussion:**

DPSCs showed the ability to regenerate nerve tissue and brain ganglia, stimulating angiogenesis by expressing cell markers and controlling growth factors in mice, and high therapeutic potential in neurodegenerative disorders. The present study invites further research in neurological disorders, specifically strokes, to prescribe these stem cells to the human population.

## 1 Introduction

Tissue regeneration and restoration of organ function have been accompanied by the idea of stem cells [[1], [2], [3]]. Stem cells have proven to be capable of regenerating Bone Marrow (BM) [1,4], hematopoietic rehabilitee, and immune disorders [[5], [6], [7], [8]], and even inhibit tumor metastasis [9,10]. BM, umbilical cord blood, or teeth are rich sources of stem cells that can differentiate into target tissue cells [11,12]. Dental pulp stem cells (DPSCs) are a reliable source of stem cells accessible for long-term use. Research on their potential in various tissues began in 2000 [13,14]. DPSCs have been able to control the immune system in autoimmune diseases and regenerate bone and cartilage in patients with arthritis [[15], [16], [17]]. Stem Cells from Human Exfoliated Deciduous Teeth (SHEDs) play a role in neuronal cell regeneration and regulate immunity in patients with Multiple Sclerosis (MS) [18,19], introducing this type of treatment as safe and tolerable in clinical trials. Stem cells and neural regeneration that have lost their protective tissue under inflammatory and immune system attacks have been an essential step toward related therapies by suppressing the immune system [[20], [21]]. However, mesenchymal stem cells can reduce brain tissue inflammation and have appeared successful in reducing pro-inflammatory cytokines. Regenerating neural tissue with stem cells is a promising approach [22], particularly due to their high expression of surface and brain-specific markers. This technique offers potential treatments not only for nervous system diseases but also for cardiovascular conditions and BM malignancies. Notably, the potential use of this particular source of stem cells in humans has yet to be thoroughly researched, and this is linked to the limited number of animal studies. The study found that DPSCs could decrease the volume of tissue damage, regulate neuroinflammation, and enhance motor function following a stroke [23,24]. The present research is the initial comprehensive review to explore the impact of dental pulp stem cells on Central Nervous System (CNS) regeneration, aiming to provide a unified understanding of the potential treatment of these cells.

## 2 Methods

### 2.1 Protocol

This systematic study was conducted based on the accepted criteria of Preferred Reporting Items for Systematic Reviews and Meta-Analyses (PRISMA), using the Cochrane Manual for Systematic Reviewers 5.1.0 to comply with the principle of non-bias for clinical studies and reviewed the studies in terms of allocation concealment, blinding of participants, study personnel and outcome assessors. The research question for this systematic study was developed using PICO criteria. *Literature search*.

The current study assessed published studies from January 2014 to 2024 in databases such as Web of Science, PubMed, and Scopus. Furthermore, the researchers analyzed the current title within the Cochrane Database to identify similarities and explore relevant systematic studies. The keywords were determined through a search in the MeSH database. A group of two persons searched the database (first Web of Science, then PubMed and Scopus) separately according to the keywords Stem Cell, Dental Pulp Stem Cell, Neuroinflammation, Neuro regeneration, and Neuro disorders. According to the PRISMA criteria, similar and non-English items were first removed, and then both groups screened titles and abstracts. The eligible articles were given to the co-author to compile the studies. In the case of non-subscription, the first author supervised the review of the articles, resolving any issues that arose. Ultimately, 26 studies were selected, and their texts were thoroughly assessed. No articles were included through manual citation searches.

### 2.2 Eligibility criteria

**Fig. 1:**
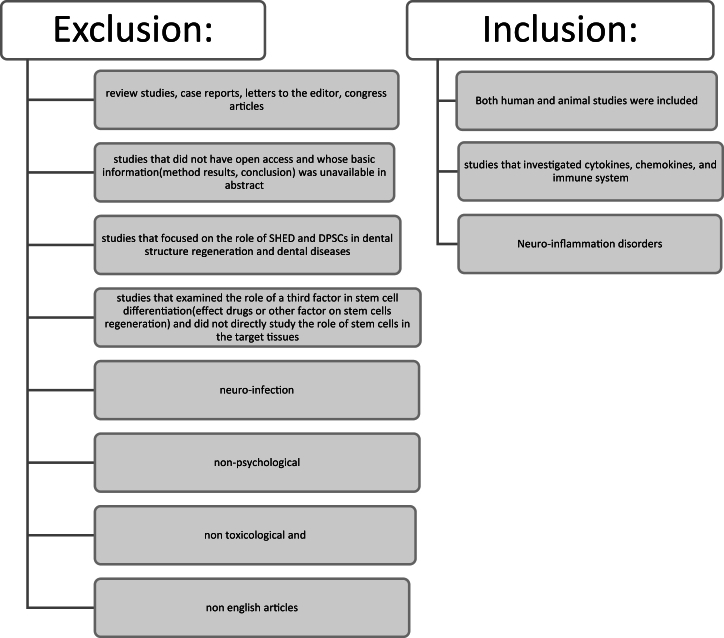
Inclusion and exclusion criteria.

All studies that investigated DPSCs and SHED were reviewed. Both human and animal studies were included. Studies that examined brain diseases based on the International Classification of Diseases 11th (ICD-11) definition were included. Studies investigating cytokines, chemokines, and immune system elements were also included, and immunity contracts were considered. In order to comprehensively investigate the role of DPSCs and SHED in the brain, all studies that examined brain diseases were included (Fig. 1).

### 2.3 Data extraction, study selection, and methodological quality assessment

At first, tissue factors, cytokines, and target tissues of DPSs were independently extracted by a group of three author, operating without coordination. The first and corresponding authors evaluated their findings and reviewed formed the summary table and thoroughly reviewed the studies based on the Cochrane tool on the risk of bias 2 (Rob 2) [25]. Then all the articles were then categorized based on these findings, that included the effect of DPSs on tissue factors, interaction with inflammatory and pro-inflammatory cytokines, and the ability to regenerate nerve tissue. Furthermore, these authors classified the articles based on the target neurological disorder (Table 2). Finally, the corresponding and first authors, a crucial step in ensuring the validity of the findings.

Key focus areas:

• First author
• Target population
• Main study aim
• Immunity factors
• Tissue markers
• Growth factors
• Main findings
• Other

## 3 Results

### 3.1 Study process

**Fig. 2:**
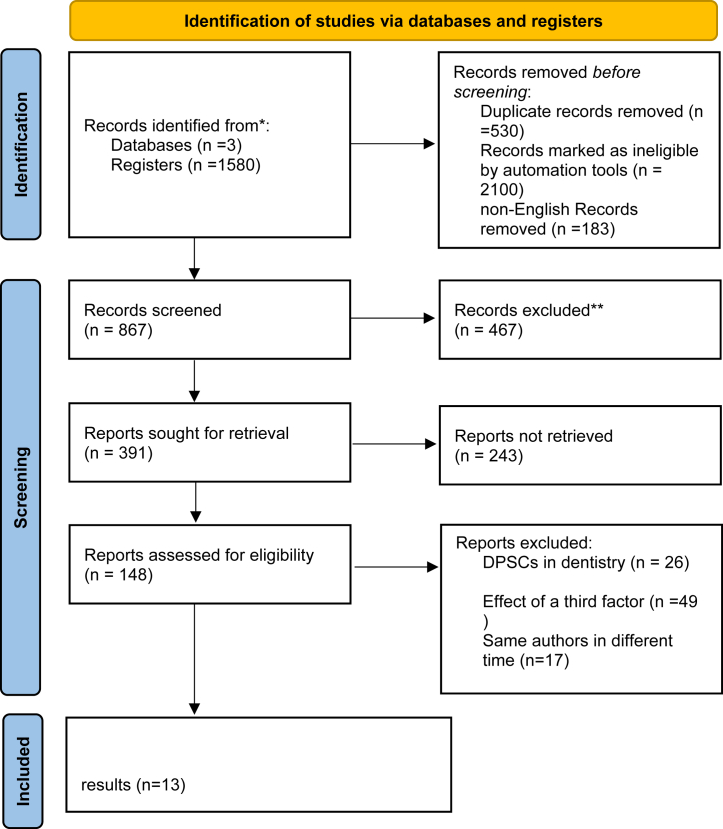
PRISMA 2020 flow diagram for new systematic reviews.

Two authors searched the keywords in the Web of Science, PubMed, and Scopus databases. A total of 1580 studies were retrieved from the three databases. Comments from the congress, Rio, and letters to the editor were excluded. After initial screening, 867 studies remained. The two-person team then reviewed the titles and abstracts, eliminating non-English, non-free, unstructured studies and those with incomplete method information. Additionally, the researchers removed duplicate titles, leaving 391 articles. The researchers further assessed these summaries based on the research criteria, finding 148 studies met the necessary eligibility (Fig. 2).

In the subsequent step, this study excluded 17 studies that were part of a research project and those investigating drug interactions on the DPS regeneration pathway. This narrowed it down to 26 articles, which the researchers examined in full, conducting a thorough review. After this comprehensive analysis, 13 articles were selected for further examination by the first and co-authors. Following a risk of bias assessment, 11 articles were deemed suitable for conclusion.

### 3.2 DPSC expresses nervous cell tissue markers

DPSCs alone can regenerate and express antigens and enzymes when transplanted with brain tissue, naturally playing an essential role in nerve cells. After transplantation, DPSCs are rich in CD44 and CD90. Expressed CD105 and CD73, as well as the factors CD29 and CD73, are the usual markers of Mesenchymal Stem Cells (MSCs). Darabi stated that DPSCs express CD 73 like other stem cells, but their tissue culture response is negative for hematopoietic markers CD 34 and CD 31. Another study mentioned that DPCs are less powerful in expressing CD14, CD19, CD34, and CD45 than in expressing CD73, CD90, and CD105 markers [[26], [27], [28], [29]]. All the factors that DPSC impacted their process are evident in Table 1. Finally, DPSCs express markers similar to other stem cells, such as MSCs, but they cannot express hematopoietic markers. They may not be transplanted in blood and BM malignancies, but they are as efficient as other fundamental sources in expressing brain markers.

**Table 1:** This table shows the immune and infectious elements related to DPSC activity. See abbreviations in terms.

*First author/Year:*	Antigen and markers	Nerve cells factors	Inflammation factors	Neural enzymes
*1. Song Li (2021)*	CD73, CD34, CD90, CD105, CD14, CD45, CD19	HMGB1, MyD88	IL‐6, IL‐1β, TNF‐α, TLR-4	NF-κB p65,
*2. Shahram Darabi (2019)*	Oct-4, CD73, CD31, CD34	MAP2,	NF160	ChAT
*3. Takeshi Tsuruta (2018)*			iNOS, IL-1β, M2 macrophage, IL-10, Lif, Ccl2	arginase-1 (Arg-1), chemoattractant protein-1
*4. Yao Liu (2022)*		NGF,	M2 Macrophage. TNF-α, IL-1β, iNOS, TGF-β1	
5. Sivapriya Senthilkumar (2023) *				
*6. Ugnė Jonavičė (2021)*		P2X4 receptor	MFG-E8	
*7. Chikako Nito (2018)*	CD29, CD73, CD90, and CD105, CD34 or CD45	VEGF	TNF-α, IL-1α, IL-1β, IL-1α, IL-1β IL-1 receptor antagonist, IL-1 IL-2, IL-3, IL-4 IL-5 IL-6 IL-10 IL-13 IL-17, IFN-γ, Granulocyte-macrophage colony stimulating factor, MIP-1α and 3α	
*8. Imran Ullah (2018)*		Microglial Iba1, NFkB,	TGF-β, TNF-α, IL-4, TLR	pAMPK, SIRT1
*9. Abdullkhaleg* *Albashari (2020)*		MAP-2, acetyl-α-tubulin	TNF-α, IL-6, Macrophages	NF-κB, IκB-α
10. Neda Eskandari (2021) *			TNF and IL-1β	
11. Chaitra Venugopal (2022) *			BCL-2	phosphorylated PI3K
*12. Ling-Yu Yang (2023)*			IL-6, IL-1β, TNF-α, TGF-β, IL-4, and IL-10	Arg-1
*13. Te-Fu Chen (2019)*	CD44, CD90, CD105	TIMP2 and TIMP 1	TGF-β	IL-1β

**Table 2:** This is summary table that provided from studies rudimentary. show terms in abbrevation.

First author/Year/Doi:	Neuro intervention	Stem cells origin	MAIN results	Other results
**Song Li (2021)**	Brain oedema, cerebral infarction, neurological impairment	Dental Pulp tissue	DPSC‐Exos inhibited the I/R‐mediated expression of TLR4, MyD88 and NF‐κB significantly, reduced the protein expression of IL‐6, IL‐1β and TNF‐α compared with those of the control both in vitro and in vivo,	DPSC‐Exos markedly decreased the HMGB1 cytoplasmic translocation induced by I/R damage
**Shahram Darabi (2019)**	Producing cholinergic neurons	hDPSCs were isolated from the human third molar,	hDPSCs were strongly positive for Oct-4 as a stem cell pluripotency marker and CD73 as a mesenchymal stromal cell marker, they were negative for CD31 as an endothelial cell marker and CD34 as a hematopoietic stem cell marker	optimal dose for the NGF was 50 ng/mL seven days after the induction when the highest percentage of expressing markers for the Cholinergic neurons (ChAT) was detected
**Takeshi Tsuruta (2018)**	Regenerate superior laryngeal nerve (SLN)	stem cells derived from human exfoliated deciduous teeth (SHED)	The number of A-β fibers in the SHED-CM group was significantly increased compared to the DMEM (−) group,The G-ratio of A-β fibers in the SHED-CM group was higher than in the DMEM (−) group,SHED-CM promoted functional nerve regeneration following the SLN lesion	Systemic delivery of the SHED-CM resulted in significantly less weight loss compared to the Dulbecco's Modified Eagles' Medium (DMEM (−)) group,SHED-CM improved dysphagia in this stage
**Yao Liu (2022)**	partial sciatic nerve ligation (PSL)	stem cells derived from human exfoliated deciduous teeth (SHED)	Intravenous administration of SHED-CM in the early, middle, and late phases of PSL mice prevented nociceptive responses, indicating the potential of SHED-CM to inhibit both development and maintenance of NP,	SHED-CM also prevented the deficits of locomotor function caused by PSL,The qPCR and immunostaining analysis revealed that SHED-CM treatment induced the anti-inflammatory M2 macrophages in injured SCN and DRG and suppressed the PSL-induced proinflammatory conditions in SCN and glial activation in Schwann cells,
Sivapriya Senthilkumar (2023) *	neurodegeneration, neuroinflammation and neuropsychiatric comorbidities in an animal model of Temporal Lobe epilepsy (TLE),	Dental pulp stem cells (DPSCs)	Our results revealed that systemic administration of DPSCs/BM-MSCs attenuated neurodegeneration, neuroinflammation, and ameliorated neuropsychiatric comorbidities	Three months following intravenous administration of DPSCs/BM-MSCs, we observed a negligible number of engrafted cells in the corpus callosum, sub-granular zone, and sub-ventricular zone,
**Ugnė Jonavičė (2021)**	EVs induced a rapid increase in intracellular Ca2+ and promoted significant ATP release in microglial cells after 20 min of treatment, therapeutic strategies targeting disease-associated neuroinflammation,	stem cells derived from human exfoliated deciduous teeth (SHED)	EVs (4 AU/mL) significantly increased Ca2+ levels in microglia, suggesting that EVs induce a rapid and transient ATP release in human microglia, EVs activate microglial migration via the P2X4R pathway, and that this effect does not depend on EV-triggered ATP release, ose association between Milk-fat globule EGF factor-8(MFG-E8) and P2X4R proteins in human microglia which is significantly promoted in cells exposed to EVs, molecular mechanisms through which EVs target human microglia that may be exploited for the development of new therapeutic strategies targeting disease-associated neuroinflammation	cilengitide significantly suppressed EV-induced migration of microglia, pretreatment with cilengitide significantly suppressed EV-induced lipid raft formation
**Chikako Nito (2018)**	Reperfusion in brain infarction, neuroinflammation during the acute phase of stroke,	Dental pulp stem cells (DPSCs)	DPSCs transplanted at 0 h after reperfusion significantly reduced infarct volume and reversed motor deficits at 24 h and 72 h recovery, DPSC transplantation significantly inhibited microglial activation and pro-inflammatory cytokine expression compared with controls at 72 h after reperfusion	DPSCs transplanted at 3 h after reperfusion also significantly reduced infarct volume and improved motor function compared with vehicle groups at 24 h and 72 h recovery, neuroprotective action of DPSCs may relate to the modulation of neuroinflammation during the acute phase of stroke,
**Imran Ullah (2018)**	Counterbalance peripheral nerve injury (PNI)-induced oxidative stress and supraspinal neuro-inflammation	Dental pulp stem cells (DPSCs)	expression of a microglial (Iba1) marker and reactive oxygen species (ROS) was lower in DPSCs and higher in DF-DPSCs, anti-inflammatory cytokine (IL-4 and TGF-β) expression was lower at 2 weeks, while it gradually increased at 8 and 12 weeks after surgery in the SNI DPSCs and DF-DPSCs groups,	DPSCs responded early and more efficiently than DF-DPSCs to counterbalance peripheral nerve injury (PNI)-induced oxidative stress and supraspinal neuro-inflammation in rat brain,
**Abdullkhaleg** **Albashari (2020)**	SCI repair	Dental pulp stem cells (DPSCs)	application of HeP-bFGF-DPSCs regulated inflammatory reactions and accelerated the nerve regeneration through microtubule stabilization and tissue vasculature, bFGF-DPSCs treatment inhibited microglia/macrophage proliferation and activation	–
Neda Eskandari (2021) *	Treat Huntington's disease (HD)	Dental pulp stem cells (DPSCs)	DPSCs treatment hampered the shrinkage of the striatum along with the inhibition of gliosis and microgliosis,	Grafting of DPSCs could repair motor-skill impairment and induce neurogenesis, probably through the secretion of neurotrophic factors and the modulation of neuroinflammatory response in HD animal models
Chaitra Venugopal (2022) *	Halting neuroinflammation and progressive neurodegeneration in the hippocampus,	Dental pulp stem cells (DPSCs)	neuroprotection revealed that DPSCs/DPSCs-CM treatment upregulated an array of hosts' endogenous neural survival factors expression, reduced pro-apoptotic caspase activity and upregulated the anti-apoptotic factors BCL-2 and phosphorylated PI3K prominently than BM-MSCs/BM-MSCs-CM,	neural crest originated DPSCs might be a better adult stem cell candidate for treating neurodegenerative diseases
**Ling-Yu Yang (2023)**	Microcirculation impairment treat	Dental pulp stem cells (DPSCs)	DPSC-CM treatment also alleviated the expressions of water channel protein aquaporin-4 (AQP4) and pro-inflammatory cytokines, and enhanced the expressions of anti-inflammatory factors in the cortical region, DPSC-CM treatment reduced hemolysate/SAH-patient CSF-induced astrocyte swelling and promoted M2 microglia polarization, partially through IGF-1/AKT signaling,	DPSC-CM treatment decreased the brain water content, improved microcirculation impairment and enhanced functional recovery at 24 h post-SAH, all the beneficial effects of DPSC-CM were abrogated after treatment with IGF-1 neutralizing antibody,
**Te-Fu Chen (2019)**	Contribute to immune modulation and neurodegeneration	Dental pulp stem cells (DPSCs)	Intrathecal administration of DPSC-derived conditioned media (DPSC-CM) ameliorated aSAH-induced vasoconstriction, neuroinflammation, and improved the oxygenation in the injured brain,	aSAH-induced cognitive and motor impairments were significantly improved by this DPSC-CM administration, Antibody-mediated neutralization of IGF-1 moderately deteriorated the rescuing effect of DPSC-CM on microcirculation, Iba1-positive cells in the injured brain area, and the cognitive/motor impairments,

### 3.3 DPSC decreases ischemia by regenerating growth factors and immune factors' effects on brain tissue

Articles on cerebral ischemia have focused on animal models, revealing that DPSC can reduce the SAH. This condition involves blood collecting in the subarachnoid space, leading to ischemia, particularly the delayed type [26,29,30]. IGF decreases due to unknown reasons in ischemia, which usually positively correlates with factors such as IL-6 in this pathway. This review notices a similar situation in SAH for IGF compared to the control group [30]. IGF, TGF-b, and TIMP1/TIMP9 significantly increased Chen's study rats treated with DPSCs [[29], [30], [31]].

Metalloproteases (MMPs) and their inhibitors (TIMPs) are enzymes that play a critical role in protein catalysis. Evidence shows that MMP-9 is linked to increased cerebral ischemia [32]. By enhancing the activity of inhibitors like DPSCs (or increasing TIMPs), blood circulation can improve, aiding in the regeneration of Cerebrovascular Accident (CVA) and ameliorating ischemia [[26], [27], [28],^33^]. One study highlighted that mice treated with DPSCs did not exhibit higher oxygen tension and MVP, yet overall cerebral perfusion improved [29].

Ultimately, DPSCs enhance blood circulation by expressing growth factors and increasing TIMP1 and TIMP9 levels. This suggests that both ischemic strokes and intracerebral hemorrhages can be managed effectively in stroke patients.

### 3.4 DPSC suppresses inflammation, regenerates the nerve ganglion and cerebral tissue

TLR-4 creates a strong inflammatory response in microglia cells that DPSCs limit this protein signaling pathway and improve tissue reperfusion in ischemia [26,34]. These proteins left strong traces in inflammatory processes, specifically the selection of microglia. In BV 2 microglia mice under the influence of DPSC, inflammation was somewhat reduced. Exosomes derived from DPSC inhibited IL-6, IL-1β TNF-α, and TGF, the main factors of nerve tissue inflammation [28,35,36]. Some protein factors such as CINC-1, CNTF, IL-3, IL-10, and IL-17 are also expressed by DPSCs; the highest expression is related to vascular growth factors [28]. VEGF is crucial for promoting growth and angiogenesis, especially during embryonic development [37]. DPSCs are known to express high levels of VEGF in vitro [28]. While VEGF is not the only pathway involved in promoting angiogenesis, it is a major contributor. Research, including ELISA tests, has demonstrated that these stem cells can inhibit pro-inflammatory necrosis factors. Additionally, studies have shown that reducing infarct volume in mice leads to recovery from ischemia, highlighting the therapeutic potential of DPSC [26,28,38].

DPSCs regenerate neural tissue growth proteins and nerve-related antigens, thereby enabling the regeneration of nerve tissue in a range of disorders. Although hematopoietic markers did not change much, OCT-4 and CD-73 were increased by DPSCs [27]. These stem cells can express neural maturation factors in neural tissue culture [27,39]. Eskandari also shows the effect of these cells on the regeneration of peripheral nerves for movement, control of inflammation, and the release of neurotrophic factors [39].

DSCs enable energy release through the P2X4R/MFG-E8 mechanism to neuro tissue regeneration [40]. Prevention of inflammation, along with the regeneration of tissue markers, can justify nerve regeneration in the swallowing process [33]. Venugopal emphasizes that DPSCs have a significant superiority over other stem cells in the property of nerve regeneration [41]. These stem cells have shown the ability to regenerate nerves, especially during Blood-Brain Barrier (BBB) disruption [42].

Pro-inflammatory factors can be found in nervous system disorders, specifically degenerative disorders. Managing inflammatory activity in the central nervous system holds therapeutic potential for restoring nervous tissue health. Spekker states that neuropeptides are responsible for releasing pro-inflammatory factors TNF and IL-6. Hidradenitis suppurativa (HS) is the main pathology of cognitive and functional disorders mainly related to epilepsy, primarily temporal epilepsy, with the release of IL-1b, TNF, and IL-6. Multiple Sclerosis (MS) is an auto-inflammatory disease based on the increase of TNF-a and the decrease of TGF-b. Ultimately, focusing on pro-inflammatory factors and cytokines of the immune system is useful in the prognosis, diagnosis, treatment, and rehabilitation of nerve cells and neurological disorders.

## 4 Discussion

In the pathogenesis of stroke, there are two main types: ischemic and hemorrhagic. One common form of cerebral hemorrhage that can result in a stroke is SAH [[43], [44], [45]]. In interventional animal studies, DPSCs do not strongly show the ability to increase hematopoietic markers; in this case, they leave the competition to MSCs obtained from other sources [27,46]. However, DPSCs enhance cerebral blood circulation and tissue perfusion in the brain. Undeniably, treating brain tissue ischemia should be expected from the general characteristics of stem cells. Nevertheless, these cells (DPSCs) express several antigens that regenerate CNS tissue, such as blood vessels and nerve tissue.

When damaged capillaries in SAH are accompanied by a decrease in the volume of incoming blood loading [47], the stimulation of angiogenesis for new capillaries by DPSCs through an increase in the expression of angiogenic factors and tissue antigens could potentially lead to a reduction in blood pressure and an improvement in blood circulation within the brain tissue. In the present studies, articles pointed to the role of DPSCs in hemorrhagic stroke, which was strengthened by the effect of DPSCs on angiogenic factors (VEGF). Stem cells have the remarkable ability to form various tissues, making them suitable for applications in neural, liver, and diabetes treatments [[48], [49], [50], [51]]. Additionally, DPSCs can positively impact cerebral blood circulation. Besides, in this study, cerebral blood pressure did not change under the influence of DPSCs, but capillary blood circulation induced by injection of DPSCs through the foramen magnum was significantly associated with improved blood circulation. This feature of DPSCs in the management of disorders leading to stroke provides a strong potential for prevention and even rehabilitation after stroke. BBB disruption is a common and dangerous condition after stroke [52,53], mainly resulting in inflammatory pathways that cause a degeneration process through the PI3K/Akt signaling pathway [53,54]. Regulation of the AKT signaling pathway gives stem cells the therapeutic potential to enable activation of the eNOS-Sirt1 pathway to prevent ischemic stroke [30]. DPSCs revealed their capability in just one study, demonstrating the potential to repair and reduce BBB leakage. However, the exact mechanism behind this process remains unclear. In stroke, we are straightened by inflammations involving interleukins (especially IL-6 and IL-1) and immune cytokines, and inflammatory factors increase and complicate subsequent tissue regeneration [[55], [56], [57]]. In addition to tissue regeneration, stem cells use the immune system for maximum regeneration, suppress TNF-A, TGF, and other necrotic factors, and reduce IL-6 and IL-1-b (based on the present results). These cells stimulate NGF, MMP, and VEGF factors to regenerate nerve tissue, especially ganglia, and increase angiogenesis.

Compared to MSCs and NSCs, DPSCs also express tissue markers. Though they may not be as efficient as MSCs in expressing blood markers, they are exceptional in nerve tissue regeneration [26,58,59]. They effectively manage growth factors, provide ample stimulation, prevent ischemia, increase perfusion, suppress pro-inflammatory factors, and limit cytokines. The primary difference between DPSCs and other stem cells lies in the challenges associated with their extraction. DPSCs show a better advantage in being autologous and obtained from self-tooth.

Pro-inflammatory factors can be found in nervous system disorders, especially degenerative disorders [[60], [61], [62]]. Controlling the inflammatory activity in the CNS is considered to have therapeutic potential to restore the nervous tissue perfectly. Spekker states that neuropeptides are responsible for releasing pro-inflammatory factors TNF and IL-6 in CNS [63]. Even in epilepsy, HS is the main pathology of cognitive and functional disorders that are specifically related to TLE, releasingIL-1b, TNF, and IL-6 [64,65]. MS is known as an auto-immunity disease based on the increase of TNF-a and the decrease of TGF-b [66,67].

Eventually, focusing on pro-inflammatory factors and cytokines of the immune system is useful in the prognosis, diagnosis, treatment, and rehabilitation of nerve cells and neurological disorders. These cells help reduce the levels of IL-6, IL-1β, and TNF-α, as well as the activity of TLR-based signaling pathways through DPSC-derived exosomes. TLRs are crucial in the signaling pathways of TNFs during inflammation [26], including brain tissue inflammation, which DPSCs help to control. DPSC is known as a source of stem cells that has yet to be discussed, so all the study procedures were limited to animal and implanted articles. Human studies on DPSC may answer the problems of treatment with stem cells, which have faced limitations in this field. Although DPSCs may not be a complete substitute for other MSCs in some cases, they have demonstrated remarkable effectiveness in regenerating nerve tissues and microglia. The STAIR guideline [68] emphasizes the importance of restoring perfusion and regenerating damaged tissue so that DPSCs can play a crucial role in future treatments. Meanwhile, DPSCs showed the ability to regenerate nerve tissue and brain ganglia and stimulate angiogenesis by expressing cell markers and controlling growth factors in mice. These types of stem cells can control interleukins and pro-inflammatory factors to restore tissue and reduce inflammation. DPSCs regenerate nerve tissue, reduce ganglion inflammation, improve BBB dysfunction after stroke, and efficiently control brain and vascular growth factors. DPSCs show a high therapeutic potential in neurodegenerative disorders, leading to further articles and treatments. This may be the starting point for an easier and less expensive treatment for MS, stroke, and degenerative disorders such as Parkinson's.

## 5 Conclusion

Stem cells derived from teeth possess the capacity to regenerate delicate tissues like nerve tissue, contributing to nerve regeneration alongside MSC and NSC. Despite being conducted on animals, this research has shown highly encouraging results regarding nerve and nerve tissue regeneration. We encourage additional studies in the realm of neurological conditions, particularly strokes, to explore the potential use of these stem cells in humans.

## Ethics approval and consent to participate

Review and approval by an ethics committee was not needed for this study because the present study is a systematic review that held under neurology department of Hormozgan university medical science supervision. This investigation not included patient consent.

## Consent for publication

Not applicable.

## Availability of data and materials

All data generated or analyzed during this study will be made available on reasonable request.

## Funding

This research received no specific grant from any funding agency in the public, commercial, or not-for-profit sectors.

## CRediT authorship contribution statement

**Ali Rahnama Sisakht:** Writing – review & editing, Data curation. **Zahra Tavasouli:** Validation. **Ahmad Negahi:** Writing – original draft. **Seyed Alireza Hosseini:** Conceptualization. **Mohammad Satarzadeh:** Writing – original draft.

## Declaration of competing interest

The authors declare that they have no known competing financial interests or personal relationships that could have appeared to influence the work reported in this paper.

## Abbreviation

BM: Bone marrow
DPSC: Dental pulp stem cells
SHED: Human deciduous tooth stem cells
PICO: Problem/Population, Intervention, Comparison, Outcome
CNS: central nerves system
aSAH: aneurysmatic subarachnoid hemorrhage
IGF: Insulin-like growth factor
IL: Interleukin
TGF: Tumor Growth Factor
TIMP: Tissue Inhibitor of Metalloproteinase
MMP: Matrix Metalloproteinase
MVP: Mitral Valve Prolapse
TLR: Too; Like Receptor
TNF: Tumor Necrosis Factor
CINC: Cytokine-Induced Neutrophil Chemoattractant
CNTF: Ciliary Neurotrophic Factor
VEGF: Vascular Endothelial Growth Factor
ELISA: Enzyme Linked Immunosorbent Assay
OCT-4: Octamer-Binding Transcription Factor 4
MFG-E8: Milk fat globule-epidermal growth factor 8
BBB: Blood-Barain Barrier
HC: Hippocampal Sclerosis
MSC: Mesenchymal Stem cell
eNOS: Endothelial Nitric Oxide Synthase
NGF: Nerve Growth Factor
NSC: Neural Stem Cell
TLE: Temporal Lobe Epilepsy
SATIR: Stroke Therapy Academic Industry Roundtable
HMGB1: High Mobility Group Box 1
NF-κB: Nuclear Factor Kappa B
MyD88: Myeloid Differentiation Primary Response Protein 88
MAP2: Microtubule-Associated Protein 2
NF160: Neurofilament 160
ChAT: Choline Acetyltransferase
iNOS: Inducible Nitric Oxide Synthase
Lif: Leukemia Inhibitory Factor
Ccl2: Chemokine C–C motif Ligand
GDNF: Glial Cell-Derived Neurotrophic Factor
NGF: Nerve Growth Factor
TGF-β1: Transforming Growth Factor-β1
MFG-E8: Globule-Epidermal Growth Factor VIII
IFN: Interferon
GM-CSF: Granulocyte-Macrophage Colony Stimulating Factor
MIP: Macrophage Inflammatory Protein
AMPK: AMP-activated protein kinase
MAP: Microtubule-Associated Protein
TIMO: Tissue Inhibitor of Metalloproteinases
AQP4: Aquaporin-4
PNI: Peripheral Nerve Injury
PSL: Partial Sciatic Nerve Ligation
